# Tying surgeon's knot: a locking variation

**DOI:** 10.1308/rcsann.2024.0069

**Published:** 2026-01-20

**Authors:** BMH Liang

**Affiliations:** Hong Kong SAR, China

## Background

When tying the surgeon’s knot, an easy-to-remember ‘plain’ version is (i) to coil the long suture end in consistent patterns and (ii) to switch the sides of the long and the short suture ends (from near side to far side, from far side to near side) simultaneously for each ‘half knot’. However, the first ‘half knot’ may sometimes fail to hold for further tying.

## Technique

In certain clinical situations, some clinicians still use the surgeon’s knot but add a ‘locking’ step after tying the first ‘half knot’. To lock, pull one of the suture ends to the opposite side first while maintaining tension on both suture ends. Video 1 gives an example of using the long suture end to lock. In this example, the first ‘half knot’ alone fails to hold, whereas adding the locking step can hold the knot temporarily. Here, the grip of the long end is released to show that such temporary locking alone can hold the knot. In practice, there is no need to release and regrip the long end.

After the locking step, both the long and the short suture ends are on the same side. When tying the second ‘half knot’, to avoid forming the granny knot pattern, the long suture end should be coiled as if it was before applying the locking step.

## Discussion

This additional step is easy to learn and to apply, but is not common in textbooks. It is, however, inferior to other techniques for tying against higher wound tension.

## Supplementary Information

The online version contains supplementary material available at https://doi.org/10.1308/rcsann.2024.0069.

